# Soybean Resistance to Soybean Mosaic Virus

**DOI:** 10.3390/plants9020219

**Published:** 2020-02-08

**Authors:** Kristin Widyasari, Mazen Alazem, Kook-Hyung Kim

**Affiliations:** 1 Department of Agricultural Biotechnology, College of Agriculture and Life Sciences, Seoul National University, Seoul 08826, Korea; kristin@snu.ac.kr; 2 Plant Genomics and Breeding Institute, College of Agriculture and Life Sciences, Seoul National University, Seoul 08826, Korea; m.alazem@snu.ac.kr; 3 Research Institute of Agriculture and Life Sciences, College of Agriculture and Life Sciences, Seoul National University, Seoul 08826, Korea

**Keywords:** extreme resistance, *R*-gene resistance, soybean, soybean mosaic virus

## Abstract

Soybean mosaic virus (SMV) occurs in all soybean-growing areas in the world and causes huge losses in soybean yields and seed quality. During early viral infection, molecular interactions between SMV effector proteins and the soybean resistance (R) protein, if present, determine the development of resistance/disease in soybean plants. Depending on the interacting strain and cultivar, R-protein in resistant soybean perceives a specific SMV effector, which triggers either the extreme silent resistance or the typical resistance manifested by hypersensitive responses and induction of salicylic acid and reactive oxygen species. In this review, we consider the major advances that have been made in understanding the soybean–SMV arms race. We also focus on dissecting mechanisms SMV employs to establish infection and how soybean perceives and then responds to SMV attack. In addition, progress on soybean *R*-genes studies, as well as those addressing independent resistance genes, are also addressed.

## 1. Introduction

Soybean is an important food and fodder crop which is vulnerable to infection by many viruses, but only few exhibit economic importance on soybean production [1,2,3]. These economically important viruses include soybean mosaic virus (SMV), bean pod mottle virus (BPMV), soybean vein necrosis virus, tobacco ringspot virus, soybean dwarf virus, and alfalfa mosaic virus (AMV) [3]. Infection by multiple soybean viruses, such as SMV and BPMV or AMV, has also been reported to cause greater damage than infection by a single virus [4].

SMV, from the genus *Potyvirus* and the family *Potiviridae*, causes soybean mosaic disease, a disease that greatly reduces soybean production worldwide [1,5]. SMV has a very narrow host range which is limited to six plant families: *Fabaceae, Amaranthaceae, Chenopodiaceae, Passifloraceae, Schrophulariaceae*, and *Solanaceae*. The most commonly infected hosts are *Glycine soja* (wild soybean) and *Glycine max* (cultivated soybean) [3,6]. Management of SMV is limited to the use of good agricultural practices and the development of resistant cultivars via breeding and genetic engineering [7]. Several SMV strains, however, have evolved the ability to avoid recognition by the plant R-protein and to thereby establish infections which lead to the emergence of resistance breaking SMV strains [8,9]. Hence, improving the understanding of how soybean perceives and responds to SMV infection will help the development of molecular breeding towards broad-spectrum resistance against SMV.

Soybean and SMV interact in complex ways during each step of infection. SMV passively enters plant cells through natural openings or through physical wounds caused by environmental factors or insect vectors [10]. If the host is unable to recognize the SMV effector(s), a compatible interaction is established. The severity of the resulting disease depends on the ability of the virus to hijack host proteins and suppress immune responses [11].

According to the mode of interaction between plant and viruses, resistance is often classified into recessive resistance and dominant resistance. Recessive resistance is established upon the impairment of a host factor required for virus replication, or negatively involved in resistance [12]. In contrast, dominant resistance, which leads to incompatible interaction, is triggered upon the recognition of viral effector by the host resistance (R) protein [7,10]. The incompatible interaction between soybean and SMV is characterized by the induction of salicylic acid (SA), the development of a hypersensitive response (HR), and a burst in the production of reactive oxygen species (ROS). These lead to the death of the infected cells and trap the virus at the point of infection [11,13,14,15]. SA is a hallmark in many incompatible interactions, including *Rsv1*-mediated resistance against the SMV-N avirulent strain [13,16,17]. Interestingly, abscisic acid (ABA), which antagonizes the SA effect, appears to play a critical role in the incompatible interaction between the resistance gene *Rsv3* and the avirulent strain SMV-G5H [18,19]. Both SA and ABA have been reported to positively regulate plant resistance against several viruses [in both compatible and incompatible interactions], but some viruses are able to reverse the defensive effects of ABA [19,20,21,22]. 

It is well-known that plants have evolved defense mechanisms against viruses and other pathogens. Researchers have made substantial progress in understanding the ability of plants to defend against viral pathogens [23,24,25]. In soybean, three independent loci (*Rsv1, Rsv3*, and *Rsv4*) have been characterized to confer resistance against SMV strains G1–G7 [26,27] and other resistance loci (*R*-genes: *Rsc4, Rsc5, Rsc7, Rsc8, Rsc15,* and *Rsc20*) where characterized in China to confer resistance against SMV-SCs strains [28,29,30,31,32,33]. In this review, we highlight the diversity of mechanisms underlying the soybean defense response against SMV and especially the ability of *R*-genes and other genes to perceive SMV invasion.

## 2. Biological Properties and Transmission of SMV

### 2.1. SMV Genome and Gene Function

SMV has been grouped into seven strains (G1 to G7) based on its virulence to soybean lines cultivated in the United States [34], and into 22 strains (SC1 to SC22) based on the Chinese identification system [35,36]. The SMV genome consists of a single-stranded positive-sense RNA, which is approximately 10 kb long and associated with genome-linked viral protein (VPg) bound to the 5′ end and the poly (A) tail at the 3′ end of the viral genome. Both the RNA and VPg are encapsidated in rod-shaped coat protein (CP) [6,37]. The genome encodes one large open reading frame (ORF), which is translated into a large polyprotein and subsequently undergoes a proteolytic reaction yielding 10 different functional proteins. A frameshift in the P3 cistron, the SMV genome also produces a small ORF that encodes for the 11^th^ protein with a size of 25 kDa [38]. These 11 proteins are P1, HC-Pro, P3, PIPO (a product of slippage in the P3 coding sequence), 6K1, CI, 6K2, VPg, NIa-Pro, NIb, and CP (Table 1) [38,39,40].

### 2.2. Biological and Molecular Properties of SMV Infection and Transmission

SMV replicates in the cytoplasm in virus replication complexes (VRCs) which are associated with endoplasmic reticulum (ER) [60]. P3 recruits the host elongation factor 1A (eEF1A) to initiate an unfolded protein response (UPR), an adaptive response that involves the accumulation of unfolded proteins at the ER, which in turn facilitates SMV replication [51]. VPg protein binds with eIF4E to initiate translation of the polyprotein, which is subsequently cleaved by viral proteases to produce 11 distinct functional proteins [6,61,70].

**Figure 1:**
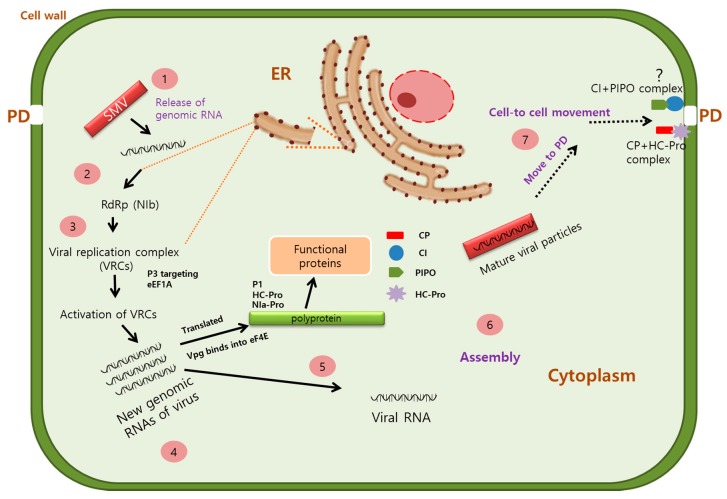
Replication and movement of soybean mosaic virus (SMV) within the cell. SMV enters the plant cell through natural openings such as the plasmodesmata (PD) or openings on the plant surface resulting from mechanical injury. Upon SMV entry, the viral genomic RNA is released and translated. Following translation of the viral proteins, virus particles assemble, and the new virus progeny move to neighboring cells. Virus movement is assisted by several functional proteins. The coat protein (CP) protects the genomic RNA, prevents degradation of viruses or virus components by host factors, and delivers the genomic RNA to PD. At PD, the proteins CI and PIPO form a CI-PIPO complex to coordinate the formation of the PD-associated structure which facilitates the intracellular movement of the virus.

Systemic infection by most plant viruses, including SMV, comprises two processes: cell-to-cell movement through plasmodesmata (PDs) and long-distance trafficking through the vascular system. PDs are essential for the intracellular trafficking of molecules required for plant life, and plant viruses have evolved to manipulate this communication system to facilitate intercellular movement [71]. The SMV MP and CP+HC-Pro complex increases PD size exclusion limits to facilitate the movement of virions into neighboring cells [72,73]. In the case of turnip mosaic virus, movement is also assisted by the PIPO protein which directs the CI protein to the PD where it forms a PIPO-CI complex [74]. This complex coordinates the formation of a PD-associated structure and facilitates the intercellular movement of the virion in the infected plants [74,75]. In addition, the 6K1 protein localizes to the cell periphery, where it is thought to have an essential function in cell-to-cell movement [55]. The viral genome is transported from the epidermal to mesophyll cells through PDs. Once the viral genome reaches the vascular bundles, long-distance trafficking of the virus is initiated (Figure 1).

SMV is a seed- and aphid-transmitted virus, and aphids uptake SMV in a non-persistent manner [47,76]. Aphid transmission depends on the interaction between HC-Pro and CP proteins. The presence of a DAG sequence in the CP facilitates the transient binding of the CP to HC-pro and is essential for the binding of virus particles to the aphid stylet and thus for aphid transmission [47,77].

## 3. Resistance Genes (*R*-Genes): Soybean Response to SMV Infection

### 3.1. NLR Gene Family-Mediated Resistance to SMV

Host resistance proteins with nucleotide-binding (NB) domains and leucine-rich repeats (LRR), shortly termed as (NLRs), represent a major class of plant immune receptors that greatly affect host–pathogen interactions [78,79]. Upon perception of pathogen effectors, NLRs trigger a cascade of downstream defense events leading to the induction of resistance against the invading viruses [80]. NLRs may represent the evolution of multifunctional single receptors, which combine sensor activity (helper) and immune signaling (executor) in a single protein, into networks of functionally interconnected receptor pairs [81]. During the perception phase, NLRs sense viral effectors, directly or indirectly, and trigger an HR in the host [81]. Most R-proteins have NLR domains located in their N-termini. NLRs are divided into two subfamilies: one with a Toll/interleukin-1 receptor (TIR) domain and the other with a coiled-coil (CC) structure [82]. TIR motifs of R-proteins are often found in dicotyledonous plants [83,84]. A comprehensive study of NLR-type R-genes led to the identification and characterization of two groups of dominant R-genes in soybean which confer resistance against SMV: 1) *Rsv* genes which confer resistance to strains G1 to G7 in the United States [85,86] and 2) *Rsc* genes which confer resistance to SMV strains SC1 to SC22 in China [35,36,87,88].

### 3.2. Rsv Genes

*Rsv1, Rsv3*, *Rsv4*, and *Rsv5* are four loci that confer resistance to different SMV strains. *Rsv1* is a highly complex locus with multiple alleles mapped to molecular linkage group (MLG) F. The dominant *Rsv1* locus is mapped to chromosome 13, and encoded candidate genes in the cultivar PI 96983 were identified as a cluster of nucleotide-binding leucine-rich repeat (NB-LRR)-type of *R*-genes [89]. *Rsv1* confers resistance to SMV strains G1 to G6 but not to G7 [90]. Phenotypes of *Rsv1*-mediated resistance against SMV strains are diverse and include extreme resistance (ER) against SMV strains G1 to G6, lethal hypersensitive response (LSHR) against SMV-G7 [52], and HR occurring on the stem, petioles, and leaf veins of plants inoculated with G2 [15].

*Rsv1*-mediated ER against most SMV strains requires multiple defense genes, including those involved in the SA and JA pathways, and may also involve specific WRKY transcription factors [17,91]. Silencing soybean orthologs of the SA-related genes *GmEDR1, GmEDS1,* and *GmPAD4,* and the JA-related gene *GmJAR1*, in the SMV-resistant soybean line L78–379, resulted in symptoms that were similar to those recorded in the susceptible control cultivar (Williams 82) in response to infection with SMV-G2 [17]. In another study, double silencing of *GmEDS1a/GmEDS1b* or single silencing of *GmPAD4* reduced pathogen-inducible SA accumulation, which further enhanced soybean susceptibility to SMV-G5 and thereby indicated the importance of SA in *Rsv1*-resistance against SMV-G5 [91]. In addition, silencing *GmHSP90* severely stunted plants and reduced the replication and movement of SMV-G2 [17]. This suggests that the chaperone HSP90 is required for Rsv1-mediated ER in response to G2, an avirulent strain of SMV [6]. 

Many WRKY transcription factors regulate the transcriptional reprogramming associated with plant immune responses and plant development [92,93]. Several reports suggest that SA-related WRKYs are actively involved in *Rsv1-*mediated resistance against SMV-G2. For example, silencing the SA-induced WRKY6 and WRKY30 in the soybean line L78–379 compromised the *Rsv1-*mediated resistance against SMV-G2 in soybean line [17,94,95].

**Figure 2:**
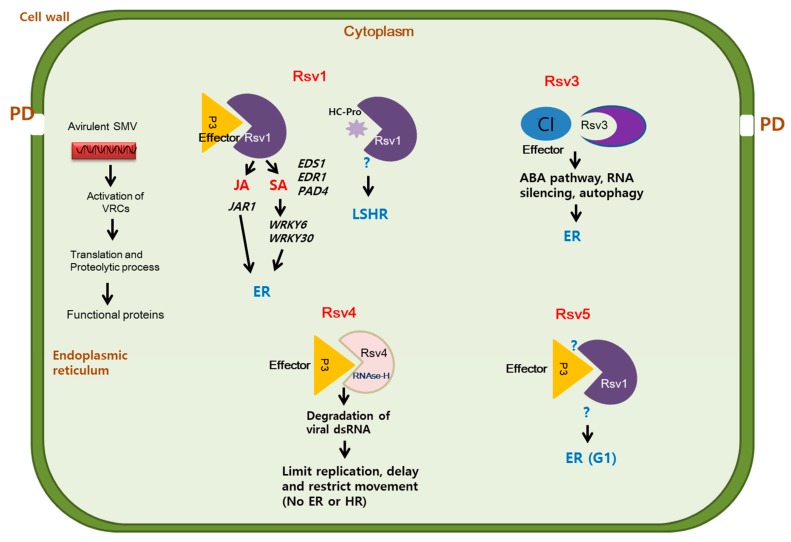
Rsv-mediated perception and resistance against SMV. Rsv1 recognizes the effectors P3 and/or HC-Pro protein; recognition of HC-Pro induces a lethal systemic hypersensitive response (LSHR), and recognition of P3 induces several host factors including *HSP90*, *EDS1*, *EDR1*, *WRKY6*, and *WRKY30*, which contribute in extreme resistance (ER) through the salicylic acid (SA) and jasmonic acid pathways. Rsv3 recognizes the CI protein and thereby induces ER where abscisic acid (ABA), and antiviral RNA silencing pathway and autophagy are triggered following infection. Rsv4 recognizes P3, which encodes dsRNAase, and targets the viral dsRNA in the replication complex leading to its degradation. The effector for Rsv5 is unknown, but the recognition results in ER in response to SMV-G1.

The P3 protein is the effector of *Rsv1*-mediated resistance, and the amino acids 823, 953, and 1112 are important for Rsv1 perception of P3 and thus for the subsequent induction of LSHR (Figure 2) [14,52]. Replacement of HC-Pro and/or P3 from avirulent strains with HC-Pro and/or P3 from virulent strains (SMV-G7 or SMV-G7d) changed the avirulent strains into virulent strains [49], suggesting that HC-Pro is also an effector for *Rsv1*-mediated resistance.

*Rsv3* is mapped to a locus between the markers A519F/R and M3Satt on chromosome 14 in the soybean molecular linkage group B2 [96]. Further investigation revealed that the *Rsv3* locus contains a family of closely related proteins with a CC motif and an LRR domain (CC-NB-LRR), suggesting that *Rsv3* encodes a member of the NLR family [96,97]. Unlike *Rsv1*, which confers resistance to a broad spectrum of SMV strains, *Rsv3* is a strain-specific resistant gene that confers ER only to SMV strains G5, G6, G7, and G5H [97] [57,98,99]. However, *Rsv3* induces necrosis and mosaic symptoms depending on the infecting strain (G1 to G4), and induces systemic mosaic symptoms upon the infection with G7H [57]. Analyses of chimeras that were constructed by exchanging fragments between the avirulent SMV-G7 and the virulent SMV-N strains showed that both the N and C terminal regions of the CI cistron are required for Rsv3-mediated ER [58]. In a different study, a single amino acid substitution in the CI region between G7H and G5H abolished ER induction in response to the chimeric G5H infection [57] (Figure 2). 

The molecular signaling involved in the Rsv3-mediated ER was elucidated using the *Rsv3*-harbouring L29 plants. Infection with G5H allows Rsv3 to recognize the CI protein, which induces several genes in the ABA pathway, including the negative regulator *PP2C3a* [18,100]. Expression of *PP2C3a* induces callous accumulation and thus restricts G5H movement at the infected points [100]. Analysis of RNA sequencing data also suggested that the *Rsv3*-mediated ER against SMV-G5H involves the antiviral RNA silencing pathway and autophagy. In addition, reduction in the expression of many genes in the jasmonic acid pathway and *WRKY* transcription factors were also observed following G5H infection on L29 plants. Interestingly, ABA can also induce resistance L29 plants against the G7H virulent strain by enhancing callous accumulation and increasing the expression of several genes involved in the antiviral RNA silencing pathway [18,19]. Future research addressing the localization of Rsv3, factors associated with Rsv3, and downstream defense signaling pathways would help us better understand the molecular mechanisms underlying *Rsv3*-mediated resistance.

The *Rsv4* locus is flanked by the microsatellite markers (SSRs) Satt542 (4.7 cM) and Satt558 (7.8 cM) [101]. Using whole genome sequencing of D26 (which carries the *Rsv4 gene)* crossed with Lee 68 (an *rsv-*null cultivar), and of V94-5152 (*Rsv4*) crossed with Lee 68 (*rsv*), it has been determined that *Rsv4* is localized in the 1.3 cM region on chromosome 2 [102]. While this region does not encode any NLR gene, several genes encoding for transcription factors were located on that region [102]. Rsv4 confers resistance to strain G1 to G7 [103]. In G2 strain, a single amino acid substitution (Q1033K) in P3 protein enabled the mutant to overcome Rsv4 resistance in the soybean cultivar V94–5152 [53]. Sequence analysis of new variants of *Rsv4-*resistance-breaking isolates revealed that these isolates contained either the Q1033K mutation or a G1054R substitution in their P3 protein [53]. The combination of Q1033K and G1054R enhanced SMV movement and symptom severity in the soybean PI 88788 (*Rsv4*) [86]. These results suggest that SMV virulence determinants in *Rsv4* cultivars are located on P3, and that Q1033K or G1054R substitution is sufficient to increase SMV virulence [53,86,104].

The strength of *Rsv4-*mediated resistance and the nature of the associated phenotypes differ between two cultivars carrying the *Rsv4* gene (V94–5152 and PI 88788) [86,103]. While SMV-N accumulated in the inoculated leaves of both cultivars, infection was much less severe in V94–5152 than in PI 88788. These results indicate that *Rsv4-*mediated resistance is affected by the genetic background of the cultivar carrying the *Rsv4* gene [86].

Given that *Rsv4* does not encode NLR genes and that *Rsv4-*mediated resistance is quite different from *Rsv1*- or *Rsv3*- mediated resistance, it was proposed that *Rsv4* belongs to a new class of resistance genes [102]. A recent study showed that *Rsv4* encodes an RNase-H family protein with dsRNA-degrading activity and interacts with the P3 protein of SMV to promote the fusion of dsRNAses with host factors involved in virus replication. This fusion result in the degradation of the viral dsRNAs [65]. 

A study on the *Rsv1* locus revealed that *Rsv1* and *Rsv1-y* are separated by 2.2 cM on chromosome 13 in the soybean cultivar York [105]. This substantial separation suggested renaming *Rsv1-y*, which confers resistance to G1 but not to G7, to become *Rsv5* [106]. The cultivar York was developed from a cross between Dorman (developed from Dunfield and Arksoy) and Hood [106]. Similar to York, Dorman and Arksoy are resistant to G1 but not to G7, suggesting that *Rsv1-y* in York came from Arksoy [106]. Pedigree analysis of 18 other soybean genotypes derived from Arksoy showed that Riple, Calhoun, and Musen have *Rsv1-y*-mediated resistance [106]. The mechanism underlying *Rsv1-y* (or *Rsv5*)-mediated resistance remains unknown (Table 2).

### 3.3. Rsc Genes

The nation-wide SMV strain identification system in China includes 22 SMV strains, designated as SC1–SC22. These strains are identified based on their response to 10 dominant soybean cultivars that are distributed in different areas in China [29,35,36]. Genes conferring resistance to SC strains are designated as *Rsc* resistance genes and mapped to the same chromosomes as *Rsv* genes (chromosome 13, 14, and 2) (Table 3) [6,35]. 

Apart from those genes indicated in Table 3, a novel locus discovered on chromosome 6 was found responsible for SMV-resistance in the soybean cultivar RN-9 [30]. The new locus was designated as *Rsc15* and was mapped to a 14.6-cM region which is flanked by two SSR markers: SSR_06_17 and BARCSOYSSR_06_0835 [30]. In RN-9, the expression of *Rsc15* during early stages of SMV-SC15 infection was highly correlated with hydrogen peroxide (H_2_O_2_) levels and peroxidase (POD) activity [30]. *Glyma06g182600* was designated as *GmPEX14* and proposed as the strongest candidate gene of *Rsc15.* It encodes a peroxisomal membrane anchor protein and has a polymorphism in the DNA/cDNA sequence alignments. Infection by SC15 increased the expression of *GmPEX14* and induced the H_2_O_2_ burst in the resistant cultivar RN-9 [30]. This suggests that peroxidases are probably involved in *Rsc15-*mediated resistance to SC15. 

In addition to single dominant resistance genes, a combination of SMV resistance genes has also been reported in China. Crosses between soybean cultivars Qihuang1 x Kefeng 1 and Dabaima x Nannong 1138-2 resulted in plants carrying *Rsc4, Rsc8*, and *Rsc14Q* genes, which confer resistance to 21 strains of SMV in China [33]. In addition, pyramiding has been used to obtain soybean lines with combinations of resistance genes. Gene pyramiding in Essex cultivar was used to generate *Rsv1Rsv3, Rsv1Rsv4*, and *Rsv1Rsv3Rsv4* isolines which are resistant to strains G1 to G7. However, the isolines *Rsv3Rsv4* was susceptible to G1 [26]. 

Given the diversity of *Rsc* genes and *Rsc* loci, and the different types of those genes, functional characterizations are required to understand the molecular bases of *Rsc-*mediated resistance against various SMV SC strains. 

## 4. Independent Host Factors Involved in Soybean-SMV Interaction

Several independent host factors with defense roles are involved in soybean-SMV interaction (Table 4). The GmeEF1A protein is hijacked by the SMV P3 protein to promote SMV replication, evidenced by inhibition of SMV accumulation in *GmEF1A*-silenced plants [51]. Mitogen-activated protein kinase (MPKs) cascades are universal signal transductions that are involved in responses to various biotic and abiotic stresses, hormone signaling, cell division and developmental processes [122]. GmMPK4, a homolog of mitogen-activated protein kinase-4 in soybean, negatively regulates SA accumulation and defense responses [123]. Silencing of *GmMPK4* resulted in stunted phenotype and cell death on the leaves and stems in the silenced plants. In addition, increase of SA and H_2_O_2_ accumulation was observed in the *GmMPK4*-silenced plants [123]. Silencing of *GmMPK6* in soybean plants caused stunted phenotypes and spontaneous cell death on the systemic leaves. Furthermore, a significant increase of pathogenesis-related (*PR*) genes and the conjugated form of SA were also observed in the silenced plants, suggesting that defense response is activated in *GmMPK6*-silenced plants even without virus infection [124]. Plants silenced with *GmMPK6* exhibited increased resistance to SMV and downy mildew infections compared with control plants. This indicates that *GmMPK6*, similar to *GmMPK4,* is a negative regulator of soybean defense responses [124]. Interestingly, transient overexpression of *GmMPK6* in *N.benthamiana* or *GmMPK6*-transgenic *Arabidopsis* showed HR-like cell death symptoms without virus infection [124]. Pathogenesis-related genes were highly induced in the transgenic *Arabidopsis* plants, suggesting a positive role of *GmMPK6* in defense response in Arabidopsis [124]. These results suggest a complexity function of *GmMPK6* as both repressor and activator of defense responses depending on the host. 

Cytochrome B5 (*GmCYB5*), a gene from a class of heme proteins associated with the endoplasmic reticulum in soybean, reduce MV-SC15 accumulation [24]. In response to infection with SMV-SC15, the expression of *GmCYB5* is upregulated to a much greater degree in RN-9 resistant cultivar than in NN1138-2 susceptible cultivar. Silencing *GmCYB5* promotes SMV-SC15 accumulation in soybean RN-9. GmCYB5 physically interacts with the P3 protein of SMV-SC15 at the cell periphery and is suggested to interfere with the role of P3 in SMV replication [24]. 

Apart from individual genes involved in SMV-soybean interaction, the antiviral RNA silencing pathway has been also reported to be involved in soybean resistance to SMV [18,19,125]. The viral replication intermediate, i.e., double-stranded (ds) RNA, is sensed by RNase type III-like enzymes called Dicer-like (DCL) proteins, which cleave the dsRNA into primary short interfering (si) RNAs of 21–24 nucleotides (nt) in length [126]. Viral-derived siRNAs (vsiRNAs) are loaded into the RNA-induced silencing complex (RISC), where they guide argonaute proteins (AGO) to cleave the viral RNA genes upon perfect complementation between vsiRNA and viral genes [126,127]. In the *Rsv3*-cultivar L29, several genes in the antiviral RNA silencing pathway were induced in response to infection by the avirulent strain G5H but showed no change or even downregulation in response to infection by the virulent strain G7H [18]. This indicates that the antiviral RNA silencing pathway contributes to the ER against G5H. In addition, ABA treatment of soybean or Arabidopsis plants induces several genes in the antiviral RNA silencing pathway, which indicates that ABA acts upstream of the RNA silencing pathway and downstream of the Rsv3 sensor protein [19,128]. Interestingly, the effect of ABA on the expression of the RNA silencing genes was stronger in Rsv3-plants then in *rsv*-null plants [128].

Micro RNAs (miRNAs) target several host genes, including the NB-LRR resistance genes, in order to regulate plant responses to different stimuli [129]. The tobacco resistance gene *N* is regulated by *miR6019* and *miR6020*, while the potato PYV resistance gene *Ry* is regulated by *miR482b* [130]. Profiling of miRNAs in the soybean cultivar Williams 82 (*rsv*), which is susceptible to SMV, and in soybean cultivar P196983 (*Rsv1*), which is resistant to SMV-G2 but susceptible to SMV-G7, revealed that *miR168* was upregulated only in the G7-infected PI96983 line and that the upregulation was associated with an LSHR [23]. *miR168* regulates expression of *AGO1*, a key RNA-slicer enzyme in the antiviral RNA silencing pathway [131]. In another example, tomato infected with turnip crinkle virus (TCV), cucumber mosaic virus (CMV), or tobacco rattle virus (TRV) exhibited decreased levels of *miR482*, which allowed the transcript levels of targeted NLRs to increase [129].

Levels of other hormones such as cytokinins and brassinosteroids and expression levels of their related genes were mildly elevated in response to infection by SMV-G5H or SMV-G7H. Cytokinins and brassinosteroids have various functions in plant growth and development and also increase plant tolerance to infection by some viruses [16,18,132].

Several soybean transgenic lines have been developed for SMV resistance (Table 5). These transgenic lines were generated either by overexpressing resistance genes or by introducing SMV genetic elements to induce pathogen-derived resistance (PDR) [133]. A recent study documented a transgenic soybean that targets the soybean endogenous gene, *eIF4E*, via an RNA interference approach [134]. The eIF4E protein is required for the accumulation of the several potyviruses, and thus is considered as a major susceptibility factor for several RNA viruses [135]. Yeast two-hybrid and bimolecular fluorescence complementation assays showed that eIF4E1 interacted with Vpg protein in the nucleus and with Nia-Pro/NIb in the cytoplasm, which suggests that eIF4E is involved in SMV replication [135]. Generation of transgenic soybean plants silenced for *eIF4E1* showed robust and broad-spectrum resistance in T1 and T2 generation against SMV-SC3, SC7, SC15, SC18, and SMV-R [135].

## 5. Conclusions and Future Perspectives

Several studies have been carried out to characterize SMV-soybean interactions leading to the identification of several *R*-genes such as the *Rsv* and *Rsc* genes as well as a few other individual genes required for resistance [15,24,35,88,102,141]. However, the molecular mechanisms underlying many of which are still lacking, and further investigations would help understand how resistant cultivars achieve resistance against various SMV strains so they can be transferred to susceptible cultivars or species [24,136]. Nonetheless, many new SMV strains have also emerged with counter-defense weapons evolved over natural selection in the field [8,9]. Their abilities to break high-specific resistance also require further investigation to determine the elements involved in resistance breaking, which in many cases involved recognition avoidance by R-proteins [8,9]. A good breeding-for-resistance strategy would aim to develop cultivars with resistance against a wide range of strains, where new molecular tools, such as CRISPR/Cas9 (which knocks out specific genes by deletion) or RNAi (which silences specific genes) can speed up the breeding program. The use of CRISPR/Cas9 in generating lines disrupted with eIF4E, a host factor required for virus replication, proved successful in generating cucumber plants with resistance to zucchini yellow mosaic virus and papaya ring spot mosaic virus-W [142]. In addition, the use of RNAi techniques to generate transgenic lines expressing fragments from SMV genes has been shown to be efficient in inducing resistance against SMV (Table 5). For instance, transgenic soybean lines expressing part of the P3 and HC-pro genes showed a stable and enhanced resistance to SMV-SC3, -SC7, -SC15, -SC18, and -R (a novel recombinant strain found in China) and have the potential to significantly increase soybean yield [137,143]. With the continuous discoveries of defense mechanisms and the implementation of new molecular tools in breeding programs, generating efficient resistant plants will be faster to achieve.

## Figures and Tables

**Table 1:** Summary of the biological functions of SMV proteins.

Protein	Function for Virus	Function for Plant
P1	Protease [41,42], Viral host range [43,44]	
HC-Pro	Long-distance movement [45], a ‘bridge’ between virion particles and aphid stylets in aphid transmission [46,47], suppression of host defense (RNA silencing) [48]	Virulence determinant [49,50]
P3	Targets host elongation factors 1A (eEF1A) to facilitate SMV replication [51]	Effector of *Rsv1* [50,52], Effector of *Rsv4* [53]
PIPO	Movement [54]	
6K1	Cell-to-cell movement [55]	
CI	Required for genome replication and movement (cell-to-cell or long-distance movement) [56]	Effector of *Rsv3* [57,58]
6K2	Formation of the virus replication complex [59,60]	
VPg	Binds specifically to eIF4E to initiate polyprotein translation [61,62]	
NIa-Pro	Proteinase [63,64]	
Nib	The catalytic subunit of RdRp [65,66,67]	
CP	A ‘bridge’ between virion particles and aphid stylets in aphid transmission [47], cell-to-cell movement, virus assembly [68,69]	

**Table 2:** Summary of *R* genes conditioning resistance to SMV.

*R* Gene	SMV Strain	Cultivar	Location	Effector	Type of *R* Gene
*Rsv1*	G1–G6 [90]	Kwanggyo Marshall Odgen PI96983 PI507389 Raiden Suweon97 Kosuzu Susumaru PI39887 Jitsuka Clifford Tousan65 Corcisa PI61944 PI61947 [107,108]	Chromosome 13	P3 [14,52] HC-pro [49]	NB-LRR-type of *R*-genes [89]
*Rsv3*	G5,G6,G7 [98,99]	Columbia Hardee Tosan140 PI 339870 PI399091 L29 [90,108]	Chromosome 14	CI [57,58]	CC-NB-LRR type or R-gene [96]
*Rsv4*	G1–G7 [103]	PI486355 V94-5152 P188788 Haman Ilpumgeomjeong KAERI-GNT-220-7 PI 398593 PI438307 Rhosa Beeson [86,108,109,110,111]	Chromosome 2	P3 [53,86,104]	Non-NLR genes (RNase-H family protein) [65]
*Rsv5*	G1 [106]	York Dorman Arksoy Riple Calhoun Musen [106]	Chromosome 13	Possibly P3	unknown

**Table 3:** Summary of the genes that confer resistance to SMV-SC strains.

R gene	SMV Strain	Cultivar	Location	Candidate Genes
*Rsc7*	SC7	Kefeng No.1 [112,113]	**Chromosome 2** Linked markers (distance): Satt266 (43.7 cM) Satt634 (18.1 cM) Satt558 (26.6 cM) Satt157 (36.4 cM) Satt698 (37.9 cM) [112] Flanking markers: BARCSOYSSR_02_0621 BARCSOYSSR_02_0632 [113]	15 candidate genes with one NBS-LRR type gene, one HSP40 gene and one serine carboxypeptidase-type gene [113].
*Rsc8*	SC8	Kefeng No.1 [32]	**Chromosome 2** Flanking markers: BARCSOYSSR_02_0610 BARCSOYSSR_02_0616 [32] Other markers: ZL-42 and ZL-52	*Glyma02g13310, Glyma02g13320, Glyma02g13400, Glyma02g13460*, *Glyma02g13470* [32] *Glyma02g121500* and *Glyma02g121600* (encoding MADS-box proteins) [114]
*Rsc5*	SC5	Kefeng No1 [28]	**Chromosome 2** Flanking markers: Bin 352 Bin353 [28]	11 candidate genes with *Glyma02g13495* as the most plausible candidate [28]
*Rsc20*	SC20	Qihuang-1 [29]	**Chromosome 13** Flanking markers: BARCSOYSSR_13_1099 BARCSOYSSR_13_1185 [29]	TIR-NBS-LRR type R genes: *Glyma13g194700* and *Glyma13g195100* [29].
*Rsc12*	SC12	Qihuang-22 [115]	**Chromosome 13** Flanking marker: Satt334 Sct_033 [115]	
*Rsc3*	SC3	Qihuang-1 [116]	**Chromosome 13** [116]	*Glyma13g25920, Glyma13g25950, Glyma13g25970,* and *Glyma13g26000* [116].
*Rsc3Q*	SC3	Qihuang-1 [117]	**Chromosome 13** Flanking markers: BARCSOYSSR_13_1114 BARCSOYSSR_13_1136 [117]	*Glyma13g25730, Glyma13g25750, Glyma13g25950, Glyma13g25970,* and *Glyma13g26000* [117].
*Rsc14Q*	SC14	Qihuang-1 [118,119]	**Chromosome 13** Flanking markers: Sat_234 Sct_033 [118] Other markers: Satt334 MY750 [119]	
*Rsc18*	SC18	Kefeng No.1 [120] Qihuang-22 [120]	**Chromosome 2** Flanking marker: BARCSOYSSR_02_0667 BARCSOYSSR_02_0670 [120] **Chromosome 13** Flanking marker: SOYHSP176 Satt334 [120]	*Glyma02g127800, Glyma02g128200* and *Glyma02g128300* [120]
*Rsc4*	SC4	Dabaima [121]	**Chromosome 14** Flanking markers: BARCSOYSSR_14_1413 BARCSOYSSR_14_1416 [31]	NB-LRR genes*: Glyma14g38510* and *Glyma14g38560* P450 family gene: *Glyma14g38580* [31]

**Table 4:** Summary of host factors or genes involved in resistance to SMV.

Host Factors	Roles in SMV Resistance	Reference
eEF1A	Targeted by P3, promotes SMV replication	[51]
*GmEDR1, GmEDS1 GmPAD4*	Induce accumulation of SA, mediated resistance against SMV	[17]
*GmHSP90*	Reduced the replication and movement of SMV-G2 (*Rsv1*-mediated resistance)	[17]
*WRKY*6 *WRKY30*	*Rsv1-*mediated resistance against SMV-G2	[17]
*GmPP2C3a*	Induces callose accumulation, restricts SMV movement	[100]
*GmPEX14*	Induces burst of H_2_O_2,_ (*Rsc15-*mediated resistance)	[30]
*GmMPK4*	Negatively regulates SA accumulation and defense response	[123]
*GmMPK6*	Repressor and activator in defense response	[124]
GmKR3	Stimulates ABA accumulation	[25]
*GmCYB5*	Targets the P3 protein to inhibit SMV accumulation	[24]

**Table 5:** Summary of SMV-tolerant cultivars.

Tolerance Cultivar		Reference
Transgenic *GmAKT2*	Alter the level of potassium, reduce the spread of SMV	[136]
RNAi-mediated silencing of SMV P3 transgenic soybean	Exhibited stable and enhanced resistance to SMV SC3 and other potyviruses.	[137]
Transgenic GmKR3	Enhances resistance against multiple viruses, including SMV-SC3, via ABA signaling	[25]
Attenuated SMV-Coat-protein mediated-resistance transgenic soybean	Highly resistant to SMV strain D and A (in Japan)	[138]
SMV-CP-RNAi transgenic soybean	Induces a functional gene silencing system and resulted in a viral-resistant phenotype.	[139]
Inverted repeat-SMV-*HC-pro* transgenic soybean	Induced RNA-mediated resistance via RNAi by targeting SMV-*HC-pro*	[140]
Soybean RNA interfere lines, silenced for eIF4E	Interferes viral replication cycles, increases broad-spectrum resistance against SMV-SC3, SC7,SC-15,SC18, and SMV-R	[134]
